# Racial Disparities in Mortality Among American Film Celebrities: A Wikipedia-Based Retrospective Cohort Study

**DOI:** 10.2196/13871

**Published:** 2019-12-10

**Authors:** Hannah Speaks, Alyssa Falise, Kaitlin Grosgebauer, Dustin Duncan, Adam Carrico

**Affiliations:** 1 University of Miami Department of Public Health Sciences Miami, FL United States; 2 Columbia University Mailman School of Public Health New York City, NY United States

**Keywords:** continental population groups, internet, mortality, race

## Abstract

**Background:**

In the United States, well-documented racial disparities in health outcomes are frequently attributed to racial bias and socioeconomic inequalities. However, it remains unknown whether racial disparities in mortality persist among those with higher socioeconomic status (SES) and occupational prestige.

**Objective:**

As the celebrity population is generally characterized by high levels of SES and occupational prestige, this study aimed to examine survival differences between black and white film celebrities.

**Methods:**

Using a Web-based, open-source encyclopedia (ie, Wikipedia), data for 5829 entries of randomly selected American film actors and actresses born between 1900 and 2000 were extracted. A Kaplan-Meier survival curve was conducted using 4356 entries to compare the difference in survival by race. A Cox semiparametric regression analysis examined whether adjusting for year of birth, gender, and cause of death influenced differences in survival by race.

**Results:**

Most celebrities were non-Hispanic white (3847/4352, 88.4%), male (3565/4352, 81.9%), and born in the United States (4187/4352, 96.2%). Mean age at death for black celebrities (64.1; 95% CI 60.6-67.5 years) was 6.4 years shorter than that for white celebrities (70.5; 95% CI 69.6-71.4 years; *P*<.001). Black celebrities had a faster all-cause mortality rate using Kaplan-Meier survival function estimates and a log-rank test. However, in a Cox semiparametric regression, there was no longer a significant difference in survival times between black and white celebrities (hazard ratio 1.07; 95% CI 0.87-1.31).

**Conclusions:**

There is some evidence that racial disparities in all-cause mortality may persist at higher levels of SES, but this association was no longer significant in adjusted analyses. Further research is needed to examine if racial disparities in mortality are diminished at higher levels of SES among more representative populations.

## Introduction

The Heckler Report, published in 1985, was the first to report statistical differences in health and mortality among races in the United States after the Civil Rights Act in 1964 [1]. Over 30 years later, there have been significant improvements to minority lifespan and health, but notable variations in mortality rate, cause of death, and prevalence of disease have persisted [2-4]. Blacks (national born and immigrants) make up a rising 13% of the US population [5]; yet, they experience the highest mortality rates for 8 of the 10 most common causes of death in the United States. In addition, they have maintained the highest mortality rate for 8 of the 10 most common causes of death as well as a 6-year and 4-year shorter life span than that of white American males and females, respectively [4].

Socioeconomic status (SES), as defined by income, education, occupation, and social status, is a product of policy and culture often studied in context with its effect on health [3,6-8]. Unlike fixed demographic variables such as gender, race, and birth date, SES measures such as education, geographic location, marital status, financial hardship, and social status can fluctuate throughout a lifetime and generally represent a dynamic measure of social influence and affluence in society [9,10]. Economic census data throughout the past century have established significant economic and educational disparities between blacks and whites in the United States. A study of US Panel Income Dynamics over 1997-2007 found that only 12.6% of those identifying as black had completed a college education compared with 32.7% of white counterparts, whereas 22.3% of blacks and 9.3% of whites had not attained a high school diploma [11]. Wealth among non-Hispanic, American whites has been found to be orders of magnitude greater than black Americans since the 1970s [12] and highly correlated to health and mortality outcomes [13-15]. Therefore, disparities in health outcomes and mortality are generally thought to be due to historical and enduring structural economic inequities such as redlining of predominantly black neighborhoods by lenders [3,7,10,11]. Recent studies quantifying racial health and mortality disparities that controlled for SES have found that differences among races are reduced but are still present [15-17]. However, there is a growing body of literature that measures not only income but also wealth and associated variables such as social standing.

Social status and wealth are understudied aspects of SES, especially in the American context. Wealth has been defined as the total sum of income, assets, and debts to quantify personal net worth. Hajat et al found a significant inverse relationship between wealth and mortality [18], which is also consistent with a previous systematic review [9]. Another study found that wealth, rather than income alone, was a more predictive measure of SES differences in international health care utilization [19]. In contrast, social status entails combinations of SES variables and public opinion of occupational groups. These measurements of occupational and social prestige use tools such as Duncan SES index and Siegel Prestige Score for common occupational groups in a population [20]. The combination of these scales indicates a higher risk of death for those in lower social positions, consistent with previous studies, but still did not examine moderation by race and gender within each level of social class as well as occupation [21]. Although these measures address wealth and occupational prestige, both associated with cultural notability or societal influence, they do not account for individuals known as celebrities and the racial disparities that may exist within these population subsets.

Celebrities are a subset of the population that have occupations associated with higher influence, notability, and wealth. Among celebrities, actors and actresses usually maintain societal standing and wealth because of the ongoing opportunities extended once fame is achieved. Owing to their top-tier social standing and relative affluence to the general population, the high societal influence of actors and actresses is hypothesized to be associated with lower mortality rates and relatively good health. Redelmeier and Singh studied trends in mortality for Oscar-nominated actors and actresses and found that the primary causes of death followed the top-reported causes of death for the general population, with an average lifespan higher than that for the general population (76-79 years) [22]. Racial diversity in American film actors has also grown steadily throughout the years, with Smith et al reporting nearly 13% black actors and 73% white actors in 2014, which closely reflects the US census demographics for the decade [23].

To date, no research has been published that observes racial disparities in mortality among American celebrities. This study sampled Wikipedia to examine racial disparities in a retrospective cohort of actors and actresses born in the 20th century. Choosing actors and actresses born in this time frame includes those that started acting at all stages of life and allows for both an ample sample size and sufficient follow-up time. On the basis of what has been found in previous studies, it is hypothesized that black American film actors and actresses experienced faster mortality rates than their white American counterparts.

## Methods

### Wikipedia Data Source and Tools

To operationalize societal standing and notability as a celebrity, Wikipedia’s list of American film actors and actresses was used as a sampling frame. Wikipedia is a nonprofit database that is maintained internationally by the public and moderated by a large group of Wikipedia editors. These editors use a set of criteria for notability, ensuring that data entered are verifiable and each subject page has enough reference and societal influence for inclusion in the online encyclopedia. If entries are under review or do not meet all criteria, they are clearly marked with a banner at the top of the page [24]. Notability is considered for every Wikipedia page and is determined by the quality and quantity of third-party sources rather than the page or article content. This editor gatekeeping method creates a unique, reliable benchmark for determining occupational prestige for actors and actresses. Wikipedia has been verified as a quality informational source by many scholars, with the most notable being *The Wisdom of Crowds* [25], which found Wikipedia to be comparable with the Encyclopedia Britannica. Others such as Vrandečić and Krötzsch have verified the reliability and value of Wikipedia as a collectively maintained database, with the diversity of authors, the number of contributors, and the ease of access continuing to improve over time, providing a sound sampling frame [26,27].

Microsoft Excel was used for data entry, observation randomization, and creation of the final dataset file. SAS 9.4 (SAS Institute 2013) was used for descriptive statistics and both survival curve estimates using LIFETEST and PHREG procedures for the Kaplan-Meier curve and Cox semiparametric logistic regression curve, respectively.

### Creating a Dataset

A dataset of URLs for US film actors and actresses was created by importing the HTML page links from the Wikipedia category pages for American film actors and actresses. These data are open source under the Creative Commons Attribution-Share-Alike License. On May 31, 2017, 12,164 entries with actor names and the Wikipedia page URL were imported from Wikipedia, formatted into a comma-separated values (CSV) dataset using a general text editor program, and imported into Microsoft Excel 2016 for randomization and data entry. The randomized data were then split among 3 people for data entry for each observation, and once there were at least 5500 entries, the Excel datasets were merged into 1 CSV file and imported into SAS for data cleaning and analysis. To check accuracy and inter-rater reliability, 200 random entries from each of the 3 coding individuals were checked for accuracy, particularly for consistent coding of race.

**Figure 1:**
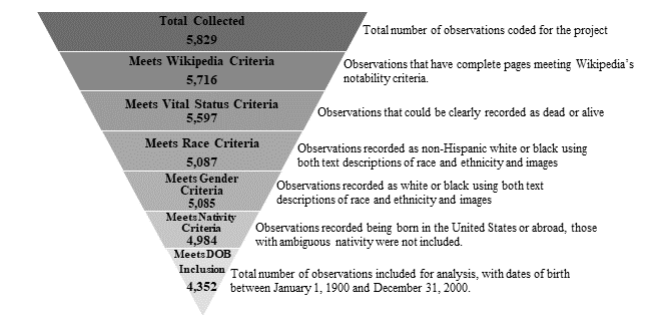
Participant selection based on inclusion criteria. DOB: date of birth.

For each film celebrity entry, a total of 6 variables were collected: gender, race, age, US nativity (yes or no), mortality status (dead, alive, or unknown), and cause of death. Wikipedia notability was confirmed by the absence of a banner indicating questionable reliability of information to confirm notability, and only those with confirmed notability were included in the final dataset. Race was recorded using the Center for Disease Control and Prevention’s definition for race based on perception of skin tone and relevant content contained in the Wikipedia entry. Collected categories were white, black, and other. Causes of death were grouped into the following categories: cardiovascular disease (CVD), suicide, accident, drug overdose, cancer, cause unknown, cause missing, and alive (no cause of death). Categories were chosen from the most common causes of death, with the addition of drug overdose and suicide because they were included in other studies focusing on similar musician populations [28,29]. CVD, suicide, and drug overdose were coded as defined by the World Health Organization, and other or unknown cause was coded as *other* [30]. The final inclusion criteria included actors that had notable Wikipedia status, were coded as either non-Hispanic white or black, had known birth and death dates (if applicable), and were born within the 20th century (January 1, 1900, to December 31, 2000). Death data were recorded with reference to May 27, 2017. In total, 5829 observations were made and 4352 (74.66%) met all inclusion criteria for study analysis (Figure 1).

### Statistical Analyses

Microsoft Excel was used for data entry, observation randomization, and creation of the final dataset file. SAS 9.4 (SAS Institute 2013) was used for descriptive statistics and both survival curve estimates using LIFETEST and PHREG procedures for the Kaplan-Meier curve and Cox semiparametric logistic regression curve, respectively. Following data collection, descriptive statistics were run to characterize the distributions and bivariate associations among variables. A student *t* test was used to compare the average age at death by race. Normality and proportionality tests were run for age at death by race and gender to select the most appropriate survival analysis methods. In this analysis, survival curves use death as an event, and the specified period is the lifespan of the celebrity. The Kaplan-Meier survival curve was created through the SAS LIFETEST procedure to plot survival for each race from date of birth to date of death, and the log-rank test was used to test statistically significant differences between each curve by race (*P*<.05). A Cox semiparametric logistic regression model was used via the SAS PHREG procedure to compare survival rates while adjusting for year of birth, gender, and cause of death. A model was built via stepwise regression to see the effect of each covariate (including race) on survival outcomes. Only covariates showing significant effect on survival rate were retained in the model, and the final regression model was then used to compare races and obtain an adjusted hazard ratio (aHR) for the all-cause mortality rate among black celebrities, with white male celebrities as the reference group.

## Results

Of the 4352 observations in the analytic sample, 1335 (30.88%) actors died during the follow-up period. Of 505 black actors observed, 405 (80.2%) were alive at the end of the follow-up period, and of 3847 white actors, 3017 (78.42%) were alive at the end of follow-up period. As shown in Table 1, most of the sample (3565/4352, 81.92%) was male, and 4187/4352 (96.21%) had US nativity. Most of the causes of death (621/1335, 46.52%) observed were recorded as dead with unknown cause, with CVD and cancer as the top 2 causes of death among our sample claiming 339/1335 (25.39%) and 251/1335 (18.80%) of those dead, respectively.

**Figure 2:**
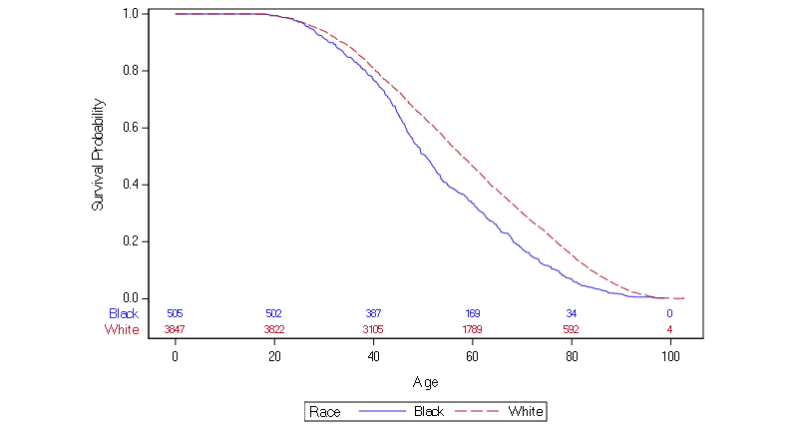
Survival probability over time by race (Kaplan-Meier plot).

**Figure 3:**
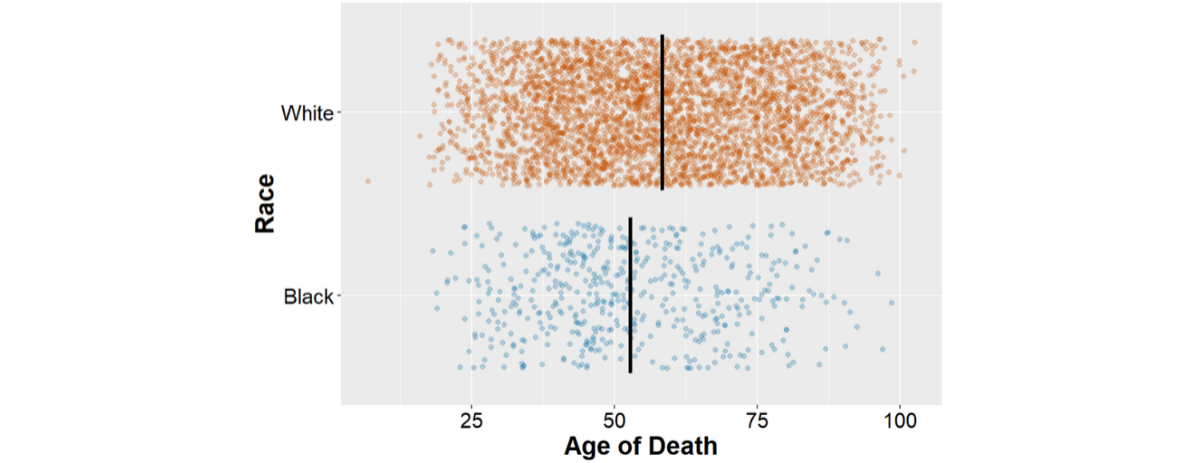
Average age of death by race.

Using the Kaplan-Meier function to compare survival rates between each race with celebrities’ genders combined (Figure 2), there was a significant difference between white and black actors when using a log-rank test for equality for each race group (*P*<.001). This was consistent with a two-sample student *t* test comparing age at death for white (70.5; 95% CI 69.6-71.4) and black (64.1; 95% CI 69.6-67.5) actors and actresses, with a significant difference in mean age at death (*P*<.001; Figure 3). On average, black actors and actresses died 6.4 years faster than their white counterparts.

Cox semiparametric logistic regression model was created via stepwise regression, with the significance threshold for inclusion set at *P*<.05. Date of birth was included as a continuous covariate with an expected hazard ratio (HR) of 1.00. The completed logistic regression model included gender (*P*=.001), cause of death (*P*<.001), and date of birth (*P*<.001) as predictors of age at death. Race was not a significant predictor of age at death using stepwise regression (*P*=.13), and when observing HRs for black actors with reference to their white counterparts, it was not found to be statistically significant (aHR 1.07; 95% CI 0.87-1.31).

**Table 1:** Results for analysis sample demographics and adjusted hazard ratios.

Study covariate		Total (N=4352), n (%)	White race (n=3847), n (%)	Black race (n=505), n (%)	*P* value	Adjusted hazard ratio^a^ (95% CI)
**Gender**					<.01	1.32 (1.120-1.56)
	Male	3565 (81.92)	3139 (81.60)	426 (84.4)		
	Female	787 (18.08)	708 (18.40)	79 (15.6)		
**Nativity**					.21	1.17 (0.91-1.50)
	American	4187 (96.21)	3702 (96.23)	485 (96.0)		
	Other	165 (3.79)	145 (3.77)	20 (4.0)		
**Vital status**					—^b^	—
	Dead	1335 (30.68)	1235 (32.10)	100 (19.8)		
	Alive	3017 (69.32)	2612 (67.90)	405 (80.2)		
**Cause of death**					<.01	1.00 (1.00-1.01)
	Cardiovascular disease	339 (7.79)	304 (7.90)	35 (6.9)		
	Cancer	251 (5.77)	227 (5.90)	24 (4.8)		
	Accident	57 (1.31)	54 (1.40)	3 (0.0)		
	Suicide	34 (0.78)	33 (0.86)	1 (0.2)		
	Homicide	15 (0.34)	12 (0.31)	3 (0.6)		
	Drug overdose	18 (0.41)	16 (0.42)	2 (0.4)		
	Cause unknown	621 (14.27)	589 (15.31)	32 (6.3)		
	Alive	3017 (69.32)	2612 (67.90)	405 (80.2)		
Date of birth					<.01	1.00 (1.00-1.00)

^a^White males were used as the reference group.

^b^Not applicable.

## Discussion

Findings from this Wikipedia study indicated that black film celebrities died 6.4 years faster than their white counterparts. Although hastened mortality among black film celebrities was observed, this association did not persist after adjusting for gender, cause of death, and date of birth. These results provide some indication that racial disparities in mortality may persist even at the highest levels of SES, and this association was no longer significant in adjusted analyses.

The discrepancies in the results between different survival comparison methods (Kaplan-Meier and Cox semiparametric logistic regression model) reflect important social changes in the representation of black actors and actresses in the American film industry during the 20th century. Black actors and actresses have historically experienced racism and other structural barriers to full participation in the American film industry, such that increased representation began only during the 1960s [31,32]. The proportion of black film celebrities in this sample was approximately 13%, which is consistent with the proportion of black Americans recorded in US census data from 2010 [4]. Nearly all black actors and actresses included in the sample were born during the second half of the 20th century. Therefore, it is likely that there has not been sufficient time to adequately assess racial disparities in mortality after adjusting for date of birth. Future research should continue to examine racial disparities in mortality among American film celebrities, and further research is also needed to elucidate if SES modifies racial disparities in mortality for black Americans.

One noteworthy finding was that only 787 (18.1%) of the random sample of 4352 Wikipedia entries were women. This may reflect the fact that many women began entering workforce in larger numbers during the last 30 years of the 20th century. However, this may also be attributable to enduring concerns related to gender bias in the American film industry [33,34]. The primary objective of this study was to examine racial disparities in a population of film celebrities with high SES. However, these stark differences in the proportional representation of women underscore the questionable generalizability of this unique sample to the broader population of the United States. Our findings clearly demonstrate that white actors appear to be the overwhelming majority in the 20th century American film industry.

Although this study underscores the potential benefits of leveraging open access sources such as Wikipedia, findings should be interpreted in the context of several important limitations. Owing to key social changes in the representation of black actors and actresses in the American film industry, it is likely that there has not been a sufficient amount of time to examine racial disparities when adjusting for date of birth. American film actors and actresses also represent a subset of celebrities from a broader population containing distinct occupational groups such as musicians and athletes. In this study, these groups were not included for several reasons. For example, inclusion of athletes introduces a fitness bias. There were also important limitations to the retrospective cohort design using Wikipedia data. Wikipedia itself has been found to have a deficit of female editors, fewer notable female Wikipedia page entries than notable male Wikipedia page entries, and biased language on female Wikipedia pages [35-37]. Most notably, some data that may have served as key confounders, such as relationship status, education level, childhood SES, and geographic residence, were not extracted [9,11,13]. Future studies with primary data collection would provide more nuanced information regarding potential confounders and effect modifiers of racial disparities in all-cause mortality.

Despite these limitations, this study provides some of the first estimates of racial disparities in mortality among American film celebrities. Results from this study have observed a 6.4-year higher mortality rate among black film celebrities, which underscores that racial disparities may persist in the United States even at the highest levels of SES. Findings support the need for further research to examine the social and psychological mechanisms that could explain the profound racial disparities in mortality experienced by black Americans.

## Abbreviations

aHR: adjusted hazard ratio
CSV: comma-separated values
CVD: cardiovascular disease
HR: hazard ratio
SES: socioeconomic status
